# 568 Mobilization, Splinting & Positioning: Increasing Nursing Participation in the Rehabilitation Plan of Care

**DOI:** 10.1093/jbcr/irad045.164

**Published:** 2023-05-15

**Authors:** Deborah Knight, Lynn Dowling, Lisa Berry

**Affiliations:** Doctors Hospital of Augusta, Augusta, Georgia; Doctors Hospital of Augusta, Augusta, Georgia; Doctors Hospital of Augusta, Augusta, Georgia

## Abstract

**Introduction:**

The foundation for long-term functional outcomes of the burn patient is laid in the Burn ICU. Nursing plays a vital role in the Rehabilitation Plan of Care for patients with burn injuries. As the member of the critical care team who is most present with the patient around the clock, the burn nurse is essential for carrying over and following through with interventions initiated by Occupational Therapy, Physiotherapy and Speech & Language Pathology. Without cohesion and partnership on the interdisciplinary burn team, the Rehabilitation Plan Care cannot succeed. In the midst of the COVID-19 pandemic, burn nursing turnover rates are higher than pre-pandemic rates. Burn Centers are relying on travel nursing contracts or novice nurses fresh out of school to provide specialized burn care. Our center developed a didactic and “hands on” learning initiative to improve collaboration between the Burn Nursing and Burn Physical Medicine departments in order to equip the critical care burn nurses to actively participate in the Rehabilitation Plan of Care.

**Methods:**

• Collaboration between Burn Nursing leadership and Burn Physical Medicine leadership to examine ABA nursing competencies pertaining to the Rehabilitation Plan of Care

• Team of PTs/OTs identified to develop curriculum based off these competencies

• Learning objectives set

• Hands on training program developed

• 8 small group training sessions completed

• Competency assessments completed

• 7 question pre- & post-testing completed to assess learning

• Opportunity for input from participants for quality improvement

**Results:**

104 Burn ICU nurses participated in the classes. 99% passed competency assessment.

Improvements between Pre- and Post- Test scores demonstrate learning of desired knowledge and skills.

Feedback assessment from RNs regarding value of course participation & ongoing need for collaborative training (most common was related to positioning & compression)

**Conclusions:**

104 burn ICU nurses participated in hands-on, didactic training related to nursing competencies related to the Rehabilitation Plan of Care. Data is now being collected regarding follow through of knowledge and skills implementation.

**Applicability of Research to Practice:**

This project showed the improvement that can be made in regards to the cooperation of burn ICU nurses with the rehabilitation plan of care.

